# LncRNA POU3F3 Contributes to Dacarbazine Resistance of Human Melanoma Through the MiR-650/MGMT Axis

**DOI:** 10.3389/fonc.2021.643613

**Published:** 2021-03-17

**Authors:** Kai Wu, Qiang Wang, Yu-Lin Liu, Zhuo Xiang, Qing-Qing Wang, Li Yin, Shun-Li Liu

**Affiliations:** ^1^Department of Burns and Plastic Surgery, People's Liberation Army (PLA) 960 Hospital, Jinan, China; ^2^Oncology Department, Shandong Second Provincial General Hospital, Jinan, China; ^3^Clinical Laboratory, Navy 971 Hospital of PLA, Qingdao, China; ^4^Pharmacy Department, Navy 971 Hospital of PLA, Qingdao, China

**Keywords:** lncRNA POU3F3, melanoma, chemo-resistance, dacarbazine, MGMT

## Abstract

**Background:** Alkylating agents are critical therapeutic options for melanoma, while dacarbazine (DTIC)-based chemotherapy showed poor sensitivity in clinical trials. Long non-coding RNAs (lncRNAs) were highlighted in the progression of malignant tumors in recent years, whereas little was known about their involvement in melanoma.

**Methods:** The functional role and molecular mechanism of lncRNA POU3F3 were evaluated on DTIC-resistant melanoma cells. Further studies analyzed its clinical role in the disease progression of melanoma.

**Results:** We observed elevated the expression of lncRNA POU3F3 in the DTIC-resistant melanoma cells. Gain-of-function assays showed that the overexpression of lncRNA POU3F3 maintained cell survival with DTIC treatment, while the knockdown of lncRNA POU3F3 restored cell sensitivity to DTIC. A positive correlation of the expression O6-methylguanine-DNA-methyltransferase (MGMT) was observed with lncRNA POU3F3 *in vitro* and *in vivo*. Bioinformatic analyses predicted that miR-650 was involved in the lncRNA POU3F3-regulated MGMT expression. Molecular analysis indicated that lncRNA POU3F3 worked as a competitive endogenous RNA to regulate the levels of miR-650, and the lncRNA POU3F3/miR-650 axis determined the transcription of MGMT in melanoma cells to a greater extent. Further clinical studies supported that lncRNA POU3F3 was a risk factor for the disease progression of melanoma.

**Conclusion:** LncRNA POU3F3 upregulated the expression of MGMT by sponging miR-650, which is a crucial way for DTIC resistance in melanoma. Our results indicated that lncRNA POU3F3 was a valuable biomarker for the disease progression of melanoma.

## Introduction

Wide multidisciplinary approaches, along with the immune checkpoint inhibitors and targeted therapies have substantially improved the survival of patients with melanoma (1). Combined treatment with alkylating agents and targeted therapies is a recommended option for metastatic melanoma, whereas the treatment with alkylating agents, such as dacarbazine (DTIC), showed a complete response rate of <5% (2, 3). Nevertheless, it was calculated that the increasing incidence of malignant melanoma and low survival rate occurred in the late-stage patients (4). It was critical to assess the mechanism of therapeutic resistance to improve the available therapies (5, 6).

Extensive studies indicate various strategies for DTIC-resistant melanoma cells (7). Among them, O^6^-methylguanine-DNA-methyltransferase (MGMT), a damage-reversal suicide enzyme, plays an important role in the chemotherapy resistance related to alkylating agents (8). The MGMT transfers an alkyl group from the O^6^-guanine of DNA to protect the cells against the DTIC-induced DNA damage. Clinical trials identified elevated MGMT expression in melanoma metastases as an indicator for DTIC-based therapy resistance (9). Therefore, further research for MGMT regulation is valuable to explore a novel strategy for reversing the chemotherapy resistance.

Recent studies on long non-coding RNAs (lncRNAs) indicated that lncRNAs played crucial roles in chromosome modification, gene transcription, and intranuclear translocation (10). However, rear studies explore the significance of lncRNA in malignant melanoma. Earlier, we screened out increased levels of lncRNA POU3F3 (also LINC01158) in DTIC-resistant melanoma cells, indicating a promising role of lncRNA POU3F3 in the acquired DTIC resistance. LncRNA POU3F3 is a 747 bp transcript located in Chr2q12.1, which shows absolute conservation among different orthologous species (11). Moreover, the elevated lncRNA POU3F3 expression was also reported in glioma (12), esophageal cancer (13), and cervical cancer (14), which was correlated with cell proliferation, migration, and invasion. Extensive research on the molecular mechanisms, including the crosstalk with miRNA, was needed for the lncRNA POU3F3-participated DTIC-resistance.

Thus, we explored the biological role of lncRNA POU3F3 in regulating DTIC-resistant melanoma cells, as well as their clinical significance in patients with malignant melanoma.

## Materials and Methods

### Cell Culture

Human melanoma cell lines, namely A375 and MV3, were obtained from the American Type Culture Collection (ATCC) (Manassas, USA). The cells were cultured in an RPMI-1640 medium with 10% fetal bovine serum (FBS). The DTIC (Sigma-Aldrich, St. Louis, USA) was dissolved in 1 M hydrochloric acid and diluted to various concentrations in a culture medium. Gradient concentration of DTIC was added in the medium for 6 months to generate the DTIC-resistant cells (A375/DTIC and MV3/DTIC).

### Cell Transfection

The pIRSE2-lncRNA POU3F3, full-length MGMT, MGMT-3′-untranslated regions (3′-UTRs), and small interfering RNAs (siRNAs) for MGMT as well as the mutant ones were synthesized in Sangon Biotech (Shanghai, China). The siMGMT were designed as reported by Veil et al. (15). The miRNA-650 mimic and the inhibitor were prepared in GenePharma (Shanghai, China). Cell transfection was performed with Lipofectamine 2000 (Invitrogen, Carlsbad, CA) and verified by quantitative real-time-PCR (qRT-PCR) assays.

### Quantitative Real-Time PCR (qRT-PCR)

Total RNA of cultured cells was prepared with the TRIzol reagent (Invitrogen, CA, USA). The AMV Reverse tTanscriptase XL (Takara Bio, Otsu, Japan) and RevertAid™ H Minus First Strand cDNA Synthesis Kit (Takara) were used for reverse transcription. The expression levels of lncRNA and micRNA were measured with the One Step SYBR Prime Script RT-PCR Kit (Takara Bio, CA, USA). The GAPDH and U6 were used as their endogenous controls. All experiments were independently repeated at least three times. The sequences of the primers are listed in the Supplementary Table 1.

### MTT Assay

Transfected cells were seeded into 96-well plates and treated with the DTIC of indicated concentration for 48 h. Cell viability was analyzed with the MTT Cell Proliferation and Cytotoxicity Assay Kit (C0009, Beyotime, Shanghai, China), which was performed according to the instructions of the manufacturer. Cell viability was measured at OD450 nm with a microplate reader.

### Colony Formation Assay

In total, 1,000 transfected cells were cultured with 12-well plates for 14 days. Giemsa-stained cell colonies were counted and photographed. All experiments were independently repeated at least three times.

### Cell Apoptosis Assay

We collected all the attached and floating cells after the indicated treatment and resuspended them in an HEPES buffer. Cell apoptosis was determined with the Annexin V-FITC/PI kit (Beyotime, Shanghai, China) as the instructions of the manufacturer, which was measured with the BD FACSAria II flow cytometer (BD Biosciences, CA, USA).

### Western Blot

A radioimmunoprecipitation assay buffer (Cell-Signaling Tech., MA, USA) was used for total protein extraction. Primary antibodies against MGMT (ab108630) and GAPDH (ab181602) were obtained from Abcam (Cambridge, USA). Western blot assays were performed as previously reported (16). The GAPDH was used as a loading control.

### Xenograft Model

In total, 12 BALB/c-nude mice were provided by the Shanghai Laboratory Animal Resource Center (Shanghai, China). The infected A375 cells (5 × 10^6^) were subcutaneously planted (six mice in each group) and grew until 100 mm^3^. The DTIC was dissolved into 0.9% NaCl solution and intraperitoneally (IP) administered (5 mg/kg weight, every 2 days) as reported by Tsubaki et al. (17). The xenograft volume was measured every 3 days with the following formula:

$$\begin{array}{l} Volume=\left(length\times width^{2}\right)/2. \end{array}$$

The mice were anesthetized and sacrificed after 15 days of DTIC treatment. The tumor tissue from each mouse was excised and photographed.

### Immunohistochemical Staining

Paraffin-embedded xenograft sections were prepared. Primary antibodies for MGMT and Bcl-2 (ab32124) were obtained for Abcam. The immunohistochemical (IHC) staining was performed with DAKO REAL EnVision K5007 (Glostrup, Denmark) according to the instructions of the manufacturer.

### Luciferase Reporter Assay

Dual-luciferase pmirGLO-lncPOU3F3-wt and mutant reporter vectors were designed as present in the schematic diagram. HEK-293 cells were co-transfected with reporters and with miR-NC or miR-650. The luciferase activity was measured with the Dual-Luciferase Reporter Assay System (Promega, Madison, WI) after 48 h.

### RNA Immunoprecipitation

The RNA immunoprecipitation of A375/DTIC cells was performed with the Imprint RNA Immunoprecipitation Kit (Sigma Aldrich, MO, USA). The immunoprecipitates were collected with anti-argonaute 2 (Ago2) or anti-IgG antibody, which were conjugated with magnetic beads. Then, qRT-PCR assays were performed for the abundance of lncRNA and miRNA in the purified RNA.

### RNA Pull-Down Assay

The miR-650-wt or miR-650-mut was transfected into A375 cells. Streptavidin-coated magnetic beads (Life Technologies, Carlsbad, USA) were used to collect the biotin-coupled RNA complex. Then, a qRT-PCR assay was performed to measure the levels of lncRNA.

### Statistical Analysis

The statistical data were presented as mean ± SD from at least three independent experiments. The comparison between two or more groups was performed with the paired Student's *t*-test or the one-way ANOVA test. Correlation between the clinical variables was analyzed with the Chi-square test. The Kaplan–Meier analysis with the log-rank test and Cox proportional hazard methods were used for the survival estimation. The value of *p* < 0.05 was considered as a significant difference.

## Results

### LncRNA POU3F3 Maintains Melanoma Cell Survival With DTIC Treatment

**Figure 1:**
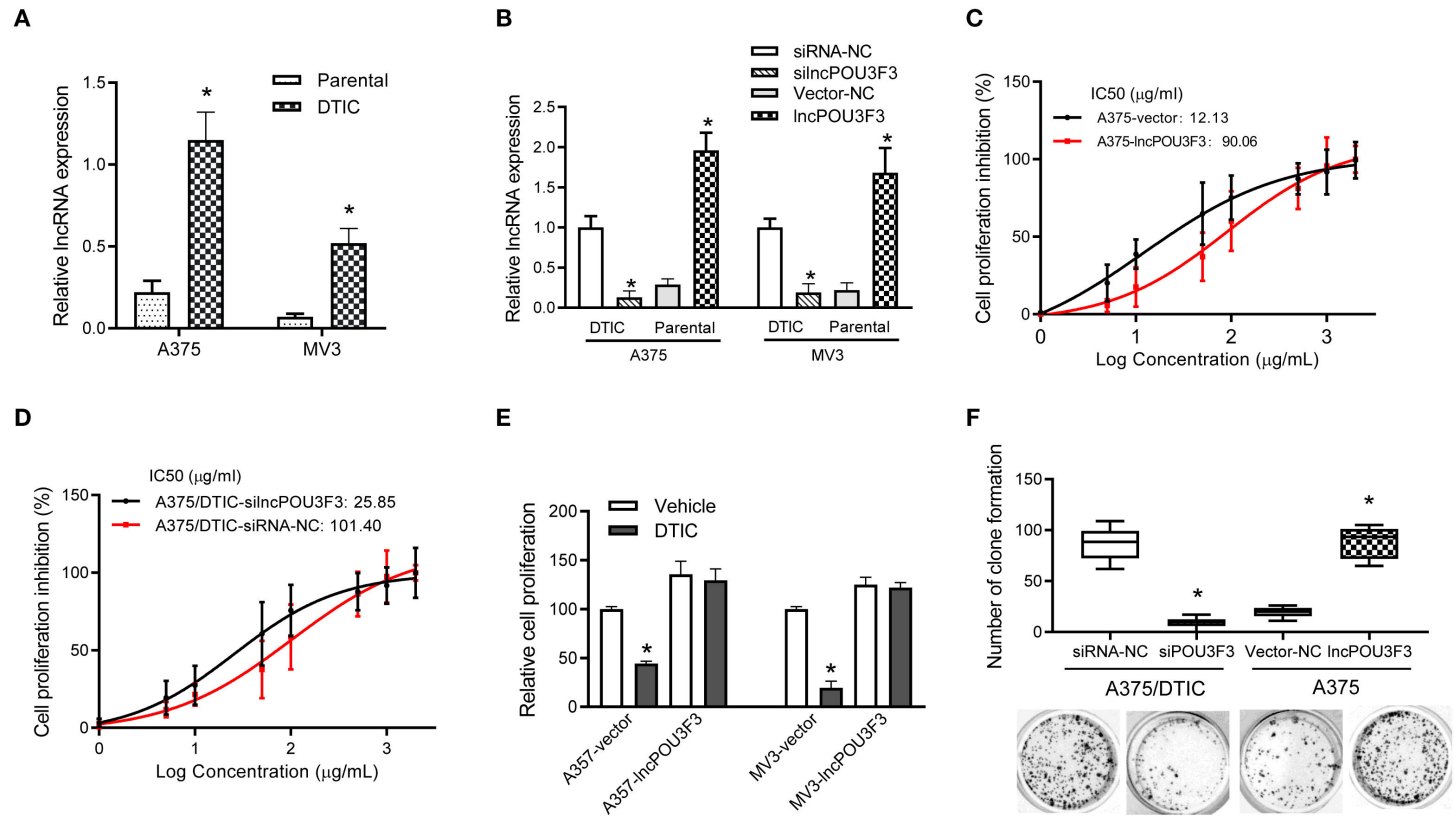
LncRNA POU3F3 maintains melanoma cell survival with DTIC treatment. **(A)** The lncRNA POU3F3 expression level was measured in DTIC-resistant melanoma cells and parental A375 and MV3 melanoma cells. GAPDH was used as the control. **(B)** The DTIC-resistant or parental A375 and MV3 cells were transfected with siRNA-NC/siRNA-lncRNA POU3F3 or Vector-NC/lncRNA POU3F3. The lncRNA POU3F3 expression levels were measured with qRT-PCR assays. **(C,D)** The transfected parental and DTIC-resistant A375 cells were treated with a series dose of DTIC (1–2,000 μg/ml) for 48 h, and cell viability was determined by MTT assays. IC50 was analyzed with the percentage of cell viability inhibition. **(E)** LncRNA POU3F3 overexpressing cells and control cells were treated with DTIC (25 μg/ml) for 3 days. The cell proliferation was detected with MTT assays. **(F)** In total, 1,000 transfected DTIC-resistant and parental A375 cells were cultured with DTIC (25 μg/ml) for 10 days. Cell colony number was compared among the groups. The data are presented as mean ± SD of three independent experiments, where *p < 0.01. LncRNA, long non-coding RNAs; DTIC, dacarbazine; siRNA, small interfering RNAs; qRT-PCR, quantitative real-time PCR.

First, the expression levels of lncRNA POU3F3 were monitored in the DTIC-resistant cells and parental cells, which showed an increased expression of lncRNA POU3F3 in the DTIC-resistant cells than in the parental ones (Figure 1A). Then, we assessed the functional role of lncRNA POU3F3 in melanoma cells. The knockdown of lncRNA POU3F3 was verified after si-lncPOU3F3 transfection in the DTIC-resistant cells (Figure 1B). Exogenous expression of lncRNA POU3F3 was performed with parental melanoma cells, in which an empty vector was used as a control (Figure 1B). The MTT assays were performed for the cell viability of the transfected cells with gradient concentrations of DTIC for 48 h. Then, we analyzed the IC50 for each cell group. An increased IC50 value was observed in lncRNA POU3F3 overexpressing cells (Figure 1C and Supplementary Figure 1A), while the knockdown of lncRNA POU3F3 induced a decrease of IC50 in the DTIC-resistant cells (Figure 1D). The transfected cells were cultured with 25 μg/ml DTIC for 3 days. The cell viability analysis indicated that the expression of lncRNA POU3F3 maintained the survival of melanoma cells with DTIC treatment (Figure 1E). Moreover, the colony formation assay showed increased cell colonies in lncRNA POU3F3 overexpressing cells with DTIC treatment, whereas the knockdown of lncRNA POU3F3 decreased the ability of cell colonies compared to the corresponding control group (Figure 1F). Similar results were also observed in the transfected MV3 cells (Supplementary Figure 1B). The results supported that lncRNA POU3F3 participated in the DTIC resistance of melanoma cells.

### lncRNA POU3F3 Contributes to MGMT-Induced DTIC Resistance

**Figure 2:**
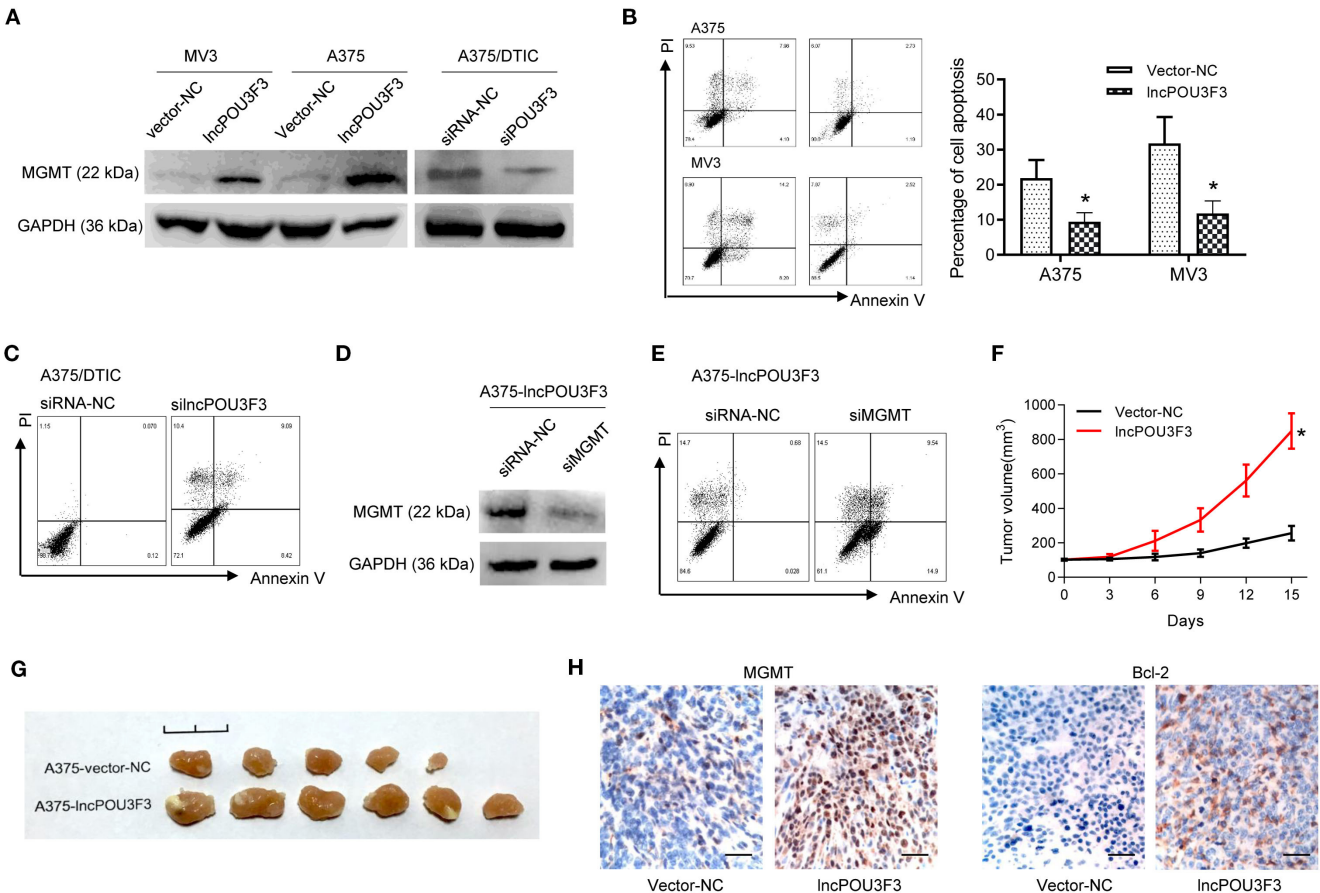
LncRNA POU3F3 contributes to MGMT-induced DTIC resistance. **(A)** Western blot assay for the MGMT expression was performed with lncRNA POU3F3 overexpressing cells, knockdown cells, and control cells. GAPDH was used as the loading control. **(B)** The transfected cells were pretreated with DTIC (A375 cells: 25 μg/ml; MV3: 10 μg/ml) for 48 h. Cell apoptosis was detected with the flow cytometry analysis. **(C)** A375/DTIC-silncPOU3F3 cells and control cells were treated with 25 μg/ml DTIC for 48 h and cell apoptosis was analyzed with a flow cytometer. **(D)** Western blot assay for the MGMT expression was performed with shMGMT transfected A375-lncPOU3F3 cells. **(E)** The transfected cells were treated with 25 μg/ml DTIC for 48 h and cell apoptosis was analyzed with a flow cytometer. The cell apoptosis data are presented as mean ± SD of at least three independent experiments. **(F)** The lncRNA POU3F3 overexpressing and controlled A375 cells were subcutaneously implanted for xenografts. DTIC (5 mg/kg) was IP administered every 2 days after the average volume of xenografts was 100 mm^3^. The volume of the xenografts was recorded every 3 days. **(G)** The volume of A375-lncPOU3F3 xenografts was compared between A375-vector and A375-lncPOU3F3 ones. **(H)** The expression levels of MGMT and Bcl-2 were determined by IHC staining with the xenografts as indicated in **(G)**, where *p < 0.01. LncRNA, long non-coding RNAs; DTIC, dacarbazine; MGMT, O6-methylguanine-DNA-methyltransferase; IP, intraperitoneally; IHC, immunohistochemical.

We further accessed the correlation of lncRNA POU3F3 and MGMT in the DTIC resistance of melanoma cells. We examined the protein levels of MGMT in transfected cells. Western blot assays showed an increased MGMT protein level in lncRNA POU3F3 overexpressing cells, whereas the knockdown of lncRNA POU3F3 reduced the MGMT levels (Figure 2A). A cell apoptosis analysis was performed with the transfected melanoma cells. The results showed that the overexpression of lncRNA POU3F3 induced a low percentage of cell apoptosis than the corresponding control cells (Figure 2B), whereas a higher percentage of cell apoptosis in the knockdown of lncRNA POU3F3 A375/DTIC cells than control cells (Figure 2C). The knockdown of MGMT was performed with lncRNA POU3F3 overexpressing cells and verified with the Western blot assay (Figure 2D). Increased cell apoptosis was observed in the MGMT knockdown cells, compared with the control group (Figure 2E).

*In vivo* assays were performed with lncRNA POU3F3 overexpressing A375 cells and empty control cells. The DTIC was administrated when the xenograft volumes were around 100 mm^3^. Increased tumor growth was observed in the tumors of lncRNA POU3F3 overexpressing cells (Figure 2F), and the xenografts showed a larger volume than the control group (Figure 2G). Moreover, the IHC staining showed an increased positive expression of MGMT and of bcl-2 in the xenografts of A375-lncRNA POU3F3 cells than in the control group (Figure 2H). These data suggested that lncRNA POU3F3 inhibited DTIC-induced melanoma cell apoptosis, which was correlated with an expression of increased MGMT.

### lncRNA POU3F3 Regulates miR-650 as a Competing Endogenous RNA

**Figure 3:**
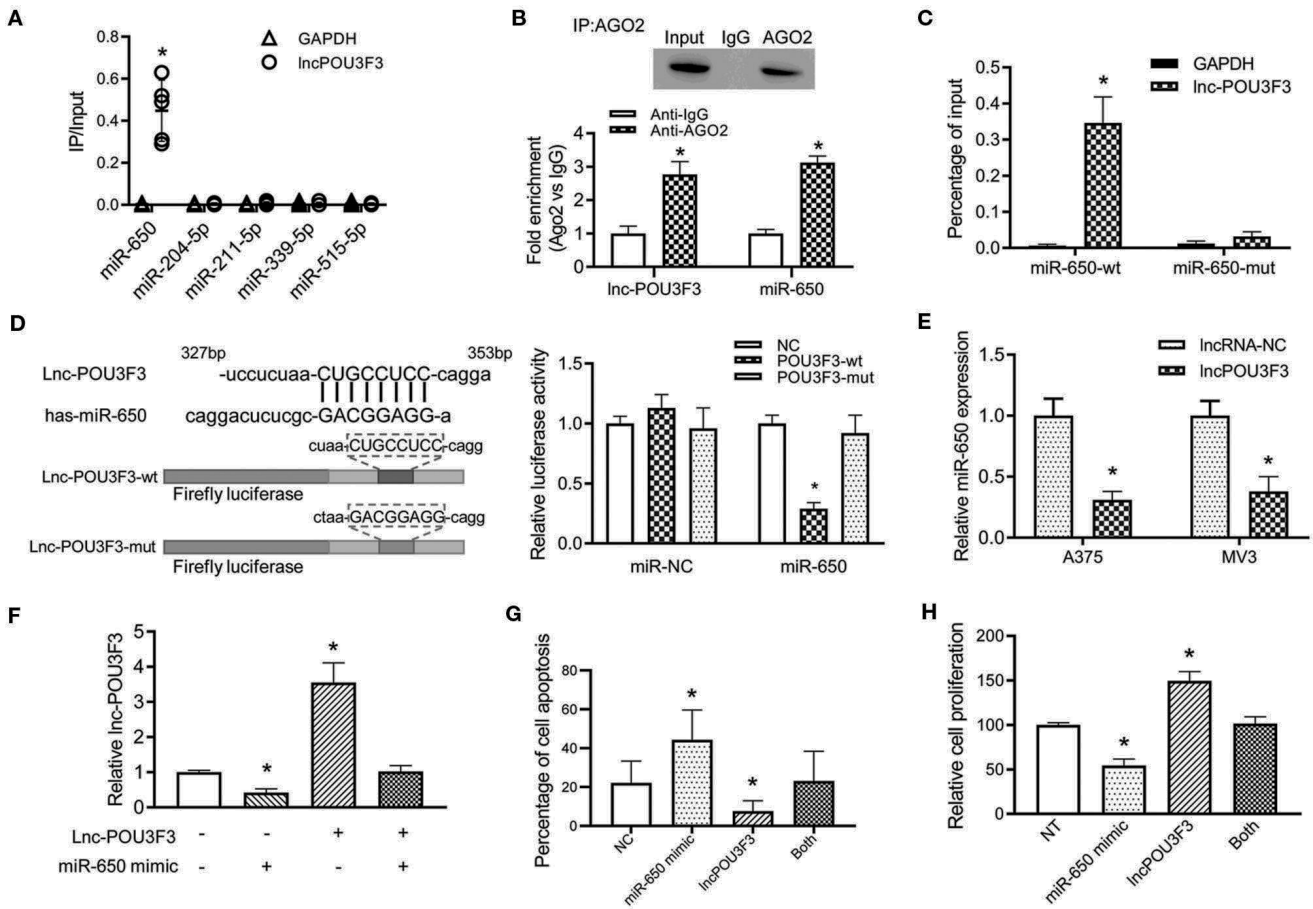
LncRNA POU3F3 regulates miR-650 as a competing endogenous RNA. **(A)** A375 cells were transfected with biotinylated miRNAs. The RNA levels of lncRNA POU3F3 and GAPDH were analyzed for the relative ratio of IP input with qRT-qPCR. **(B)** The lncRNA POU3F3 and miR-650 levels were measured in anti-AGO2 immunoprecipitates, which were analyzed with anti-Ago2 RIP assays. **(C)** The content of lncRNA POU3F3 was compared between miR-650-wt and miR-650-mut transfected A375 cells. **(D)** The predicted binding site of lncRNA POU3F3 and miR-650 was shown in the schematic diagram. Wild type and mutant lncRNA POU3F3 firefly luciferase reporters were prepared as shown in the sequence. The luciferase activity of different transfected cells was compared using a histogram. **(E)** The miR-650 expression level was analyzed in lncRNA POU3F3 overexpressing cells and vector control cells, which was measured by the qRT-PCR assay. **(F)** Relative expression levels of lncRNA POU3F3 were analyzed in the A375 cells co-infected with or without miR-650 using qRT-PCR assays. **(G)** The cell apoptosis percentage of the transfected cells **(F)** was analyzed with a flow cytometer after DTIC treatment for 48 h. **(H)** The transfected cells were treated with DTIC (25 μg/ml) for 3 days. Cell viability was detected with MTT assays. The data are presented as mean ± SD of at least three independent experiments, where *p < 0.01. LncRNA, long non-coding RNAs; DTIC, dacarbazine; IP, intrapotential; miRNAs, micro RNAs; qRT-PCR, quantitative real-time-PCR.

Previous studies indicated that a variety of lncRNAs absorbed miRNAs to regulate the transcription of the targeted mRNA (10). Then, we analyzed the reverse complementary recognition sequence of lncRNA POU3F3 to predict the target miRNAs, which was analyzed with LncBase v.2 (http://carolina.imis.athena-innovation.gr/diana_tools/web/index.php?r=lncbasev2/index). Moreover, candidate miRNAs, to regulate the MGMT expression, were also analyzed with Targetscan 7.2 (http://www.targetscan.org/vert_72/). An overlap analysis found five candidate miRNAs for further verification. An RNA pull-down analysis indicated the most relevant target miRNA of lncRNA POU3F3, which was more abundant in miR-650 than others (Figure 3A). Further analysis was performed for the interaction between lncRNA POU3F3 and miR650. RIP assays were performed with precipitated Ago2 protein. Our results showed that lncRNA POU3F3 and miR-650 were associated with the Ago2 in A375 cells (Figure 3B). Moreover, the miR-650 and lncRNA POU3F3 levels showed over 2-fold increase than the IgG control (Figure 3B). The RNA pull-down assays also indicated higher levels of lncRNA POU3F3 in miR-650-wt than in the mutant ones (Figure 3C). Further, luciferase reporter assays were conducted with the lncRNA POU3F3-wt reporter or mutant vector transfection. Our results showed lower luciferase activity in the cells transfected with the wild-type reporter and miR-650 than those transfected with miR-NC. However, cells transfected with the lncRNA POU3F3-mut reporter showed comparable luciferase activity in the cells co-transfected with miR-650 and miR-NC (Figure 3D). In addition, a decreased miR-650 level was observed in lncRNA POU3F3 overexpressed melanoma cells in the qRT-PCR assay (Figure 3E). A rescue experiment was performed with co-infection of miR-650 and lncRNA POU3F3. The qRT-PCR assay indicated that the lncRNA POU3F3 level was downregulated to a greater extent with the transfection with the miR-650 mimic (Figure 3F). Moreover, the flow cytometry analysis indicated that the miR-650 mimic transfection increased cell apoptosis with DTIC treatment, which reversed the overexpression of lncRNA POU3F3 induced the DTIC resistance in melanoma cells (Figure 3G and Supplementary Figure 1C). Moreover, the transfection of miR-650 mimic could also attenuate the cell proliferation ability in melanoma cells with DTIC treatment (Figure 3H).

### The miR-650 Regulates MGMT Expression in the Melanoma Cells

**Figure 4:**
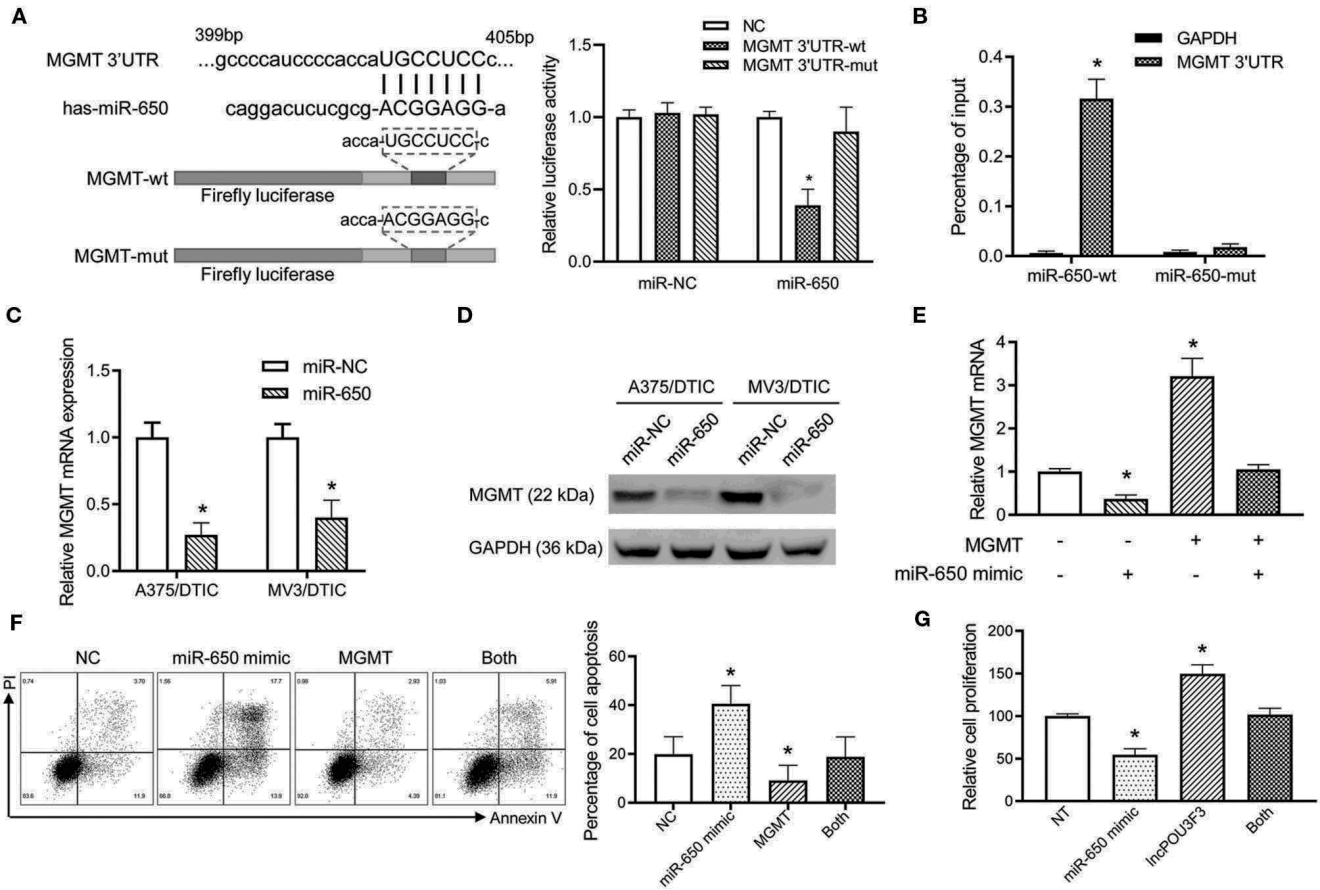
The miR-650 regulates the expression of MGMT in the melanoma cells. **(A)** The schematic diagram showed the predicted binding site of MGMT-3′UTR and miR-650. A luciferase reporter gene assay was performed for the luciferase activity in A375 cells with wild-type and mutant MGMT-3′UTR. **(B)** The MGMT-3′UTR and GAPDH RNA levels were measured with a qRT-PCR assay after the transfection of biotinylated miR-650-wt or mutant in A375/DTIC cells. The histogram showed the relative ratio to the intraperitoneal input. **(C)** The expression of MGMT mRNAs was measured with the qRT-PCR assay in miR-650 transfected melanoma cells. **(D)** The protein levels of MGMT were analyzed in miR-650 transfected cells with the Western bolt assay. **(E)** Relative MGMT mRNA expression levels were compared with the A375 cells which were transfected with the miR-650 mimic or MGMT. **(F)** The percentage of cell apoptosis of the transfected cells indicated in **(E)** was analyzed with a flow cytometer after DTIC treatment for 48 h. **(G)** The transfected cells were treated with DTIC (25 μg/ml) for 3 days. Cell viability was detected with MTT assays. The data are presented as mean ± SD of at least three independent experiments, where *p < 0.01. DTIC, dacarbazine; miRNAs, micro RNAs; qRT-PCR, quantitative real-time PCR; MGMT, O6-methylguanine-DNA-methyltransferase.

Further analysis was performed for the MGMT transcription regulation by miR-650. Luciferase reporters were prepared with a wild type or mutant MGMT 3′-UTR. The luciferase activity analysis indicated that the wild-type MGMT luciferase activity was inhibited by miR-650, while there was no significant impact by miR-NC (Figure 4A). Moreover, the mutant-type MGMT-3′UTR showed no significant change in the luciferase activity with miR-650 or miR-NC (Figure 4A). The RNA pull-down assay also indicated higher levels of MGMT 3′-UTR in wild-type miR-650 transfected with A357/DTIC cells than in the mutant-type ones (Figure 4B). Furthermore, a decreased MGMT expression was observed with the miR-650 mimic transfection in DTIC-resistant melanoma cells, which was examined with qRT-PCR assays and Western blot assays (Figures 4C,D). Moreover, MGMT plasmid and miR-650 mimics were co-transfected into A375 cells. We found that the exogenous expression of full-length MGMT was partially attenuated by the ectopic miR-650 expression (Figure 4E). The transfected cells were treated with DTIC for 48 h to analyze cell apoptosis. The results showed that the ectopic miR-650 expression increased cell apoptosis, which also attenuated the MGMT-induced DTIC resistance in melanoma cells (Figure 4F). Moreover, the infection of miR-650 mimics in melanoma cells also inhibited the cell viability of melanoma cells with DTIC treatment, which was also observed in the MGMT expressing cells (Figure 4G).

### lncRNA POU3F3 Contributes to Disease Progression in Melanoma

**Figure 5:**
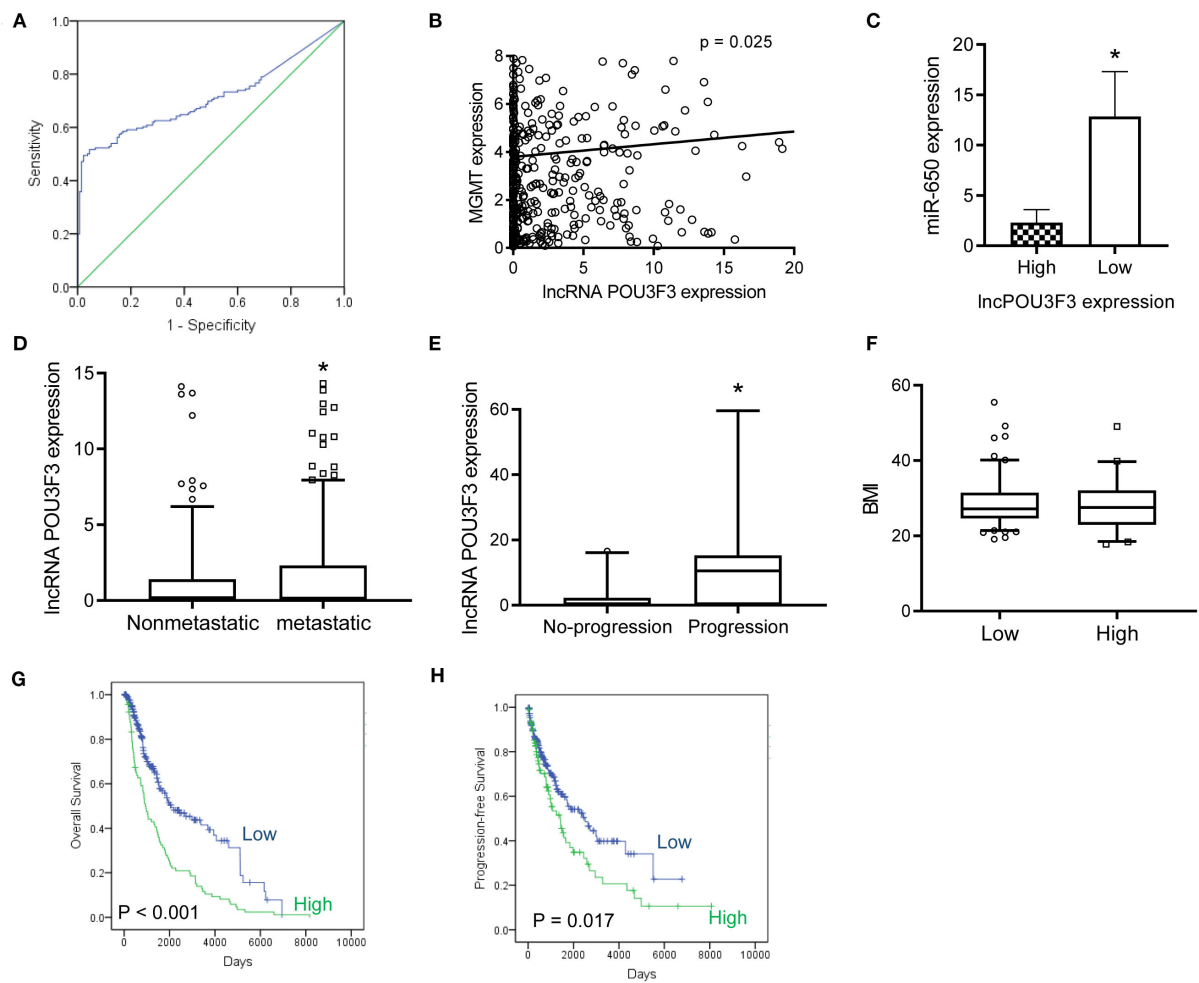
The lncRNA POU3F3 contributes to the disease progression of melanoma. **(A)** From the TCGA database, an ROC curve analysis was performed for the cut-off value of lncRNA POU3F3 in patients with melanoma (*p* < 0.01). **(B,C)** The correlation was analyzed between the expression levels of lncRNA POU3F3, MGMT, and miR-650. **(D)** The expression levels of the lncRNA POU3F3 were compared between patients with metastasis and patients without metastasis. **(E)** The lncRNA POU3F3 expression comparison was performed among 33 patients either with disease progression or without the disease progression. All patients received DTIC treatment. **(F)** The BMI was compared between the patients with high or low lncRNA POU3F3 expression, where *p < 0.01. **(G,H)** Kaplan–Meier analysis was performed for the overall survival **(G)** and progression-free survival **(H)** according to the lncRNA POU3F3 expression status. LncRNA, long non-coding RNAs; ROC, receiver operating characteristics; DTIC, dacarbazine; IP, intrapotential; MGMT, O6-methylguanine-DNA-methyltransferase.

After obtaining complete clinical information from the TCGA database, we assessed the clinical significance of the lncRNA POU3F3 expression in 309 patients with cutaneous melanoma. The ROC analysis indicated the cut-off value of lncRNA POU3F3 as 1.384 (Figure 5A). A significant positive correlation was observed in the expression of lncRNA POU3F3 and MGMT (Figure 5B). A reverse correlation was observed between lncRNA POU3F3 and miR-650 (Figure 5C). Further analysis showed an increased lncRNA POU3F3 expression in the metastatic tumors than in the localized ones (*p* = 0.01, Figure 5D). In addition, a total of 33 patients received DTIC-based chemotherapy. We found higher lncRNA POU3F3 levels in patients with disease progression than others (*p* < 0.001, Figure 5E). Further correlation analysis indicated a significant correlation between the expression of lncRNA POU3F3 and age (*p* = 0.037), whereas there was no significant correlation between gender, TNM stage, and BMI (Table 1 and Figure 5F). A univariate Cox regression analysis indicated that the TNM stage and the expression of lncRNA POU3F3 were correlated with the disease progression of melanoma (Table 2), while a multivariate analysis showed that only the TNM stage was statistically significant in the disease progression of melanoma (Table 2). The Kaplan–Meier analysis indicated a worse overall survival and progression-free survival estimation for positive patients with lncRNA POU3F3 than the others (Figures 5G,H). Our results supported that lncRNA POU3F3 was a detrimental factor for the disease progression of melanoma.

**Table 1:** Clinical characteristics of the 309 patients with melanoma.

**Characteristic**	**All**	**Low lncRNA POU3F3**	**High lncRNA POU3F3**	***p*-value**
Total	309	218	91	
**Age**				
<60	155	101	54	0.037
≥60	154	117	37	
**Gender**				
Female	111	78	33	0.936
Male	198	140	58	
**TNM stage**				
I–II	176	126	50	0.644
III–IV	133	92	41	

*TNM, tumor, node, metastasis*.

**Table 2:** Univariate and multivariate analyses of patients with the disease progression of melanoma.

**Variable**	**Univariate analysis**	***p*-value**	**Multivariate analysis**	***p*-value**
Age (≥60 vs. <60)	1.432 (0.995–2.061)	0.053		
Gender (Male vs. Female)	0.981 (0.679–1.419)	0.981		
BMI (≥24 vs. <24)	0.637 (0.371–1.096)	0.103		
TNM stage (III–IV vs. I–II)	2.699 (1.887–3.860)	<0.001	2.357 (1.429–3.890)	0.001
lncPOU3F3 (High vs. Low)	1.541 (1.079–2.202)	0.017	1.591 (0.955–2.649)	0.074

*BMI, Body mass index*.

## Discussion

LncRNA POU3F3 is located in the bidirectional promoter of POU3F3 at Chr2q12.1. The oncogenic role of lncRNA POU3F3 was verified in glioma and triple-negative breast cancer (12, 18). Recent studies identified that lncRNA POU3F3 promoted cell proliferation, invasion, and G1 cell cycle arrest *in vitro*, as well as arteriole formation *in vivo* (18–20). Our study identified an increased lncRNA POU3F3 level in DTIC-resistant melanoma cells. The gain of function assays indicated that the knockdown of lncRNA POU3F3 reversed the DTIC-resistance of melanoma cell lines, as well as decreased the ability of cell growth. The exogenous expression of lncRNA POU3F3 decreased cell sensitivity to DTIC *in vitro* and *in vivo*. Studies suggested that lncRNA POU3F3 conferred the DTIC-resistant ability of melanoma cells, which shed light to improve the therapeutic efficiency of DTIC-based chemotherapy (21).

The crosstalk between lncRNAs and miRNAs was discovered to promote malignant disease progression in various aspects of tumors, such as LINC00673/miR-150-5p in NSCLC (22). Previous studies discovered that lncRNA POU3F3 promoted the methylation of the POU3F3 gene for transcriptional repression in glioma and esophageal squamous cell carcinoma (11, 12). However, rear studies were performed for the mechanism of DTIC-related chemotherapy resistance (23, 24). In this study, a positive correlation was observed between lncRNA POU3F3 and the MGMT expression in DTIC-resistant melanoma cells *in vivo* and *in vitro*. As a dominant molecular, MGMT transports cytotoxic adducts from O^6^-guanine of DNA to avoid genomic mutation. Excessive MGMT levels induce therapeutic resistance to the treatment based on alkylating agents (25). Further studies supported that MGMT was also a predictor for clinical response to the DTIC-based therapy in patients with metastatic melanoma (26). Our study explored the mechanism of lncRNA POU3F3-induced DTIC resistance in melanoma. MiR-650 was identified and validated to mediate the transcriptional regulation of MGMT, in which the lncRNA POU3F3 sponged miR650 in DTIC-resistant cells. Endogenous competition between lncRNA POU3F3 and miR-650 regulates the expression levels of MGMT, which determines the tolerance of melanoma cells to DTIC treatment to some extent.

MicroRNAs participate in the regulation of the cellular process as post-transcriptional regulators, which directly targeted the 3′-UTRs of mRNA to repress a broad spectrum of gene expression (27). It was a ubiquitous regulation of the feedback loop between miRNAs and target genes. Various functional roles of miR-650 are regarded in different tumors, which are dependent on the target genes in a particular histological type (28). An elevated miR-650 expression is observed in hepatocellular cancer, lung adenocarcinoma, and prostate cancer, which correlates with tumor metastasis and poor prognosis (29–32). Nevertheless, the overexpression of miR-650 also indicates favorable survival estimation in chronic lymphocytic leukemia and colorectal cancer (33, 34). In this study, the tumor suppressor role of miR-650 was identified in DTIC-resistant melanoma cells. Downregulation of miR-650 by lncRNA POU3F3 facilitates the expression of MGMT in the development of DTIC resistance. MGMT is identified as a detrimental factor in alkylating agent-based chemotherapy. Further rescue experiments supported the tumor suppressor role of miR-650 in the chemotherapy for melanoma. However, there were also some limitations in the investigation mechanism. Other pathways to mediate the crosstalk between the expression of lncRNA and MGMT, including canonical signaling pathways regulation, still deserve further research.

Accumulated studies indicate that lncRNAs potentially contribute to the development of various malignant tumors (10, 22). The tumor promoting effects of lncRNA POU3F3 were reported in esophageal squamous cell carcinoma and cervical cancer (13, 14). LncRNA POU3F3 is a candidate biomarker for diagnosis and prognosis (13). We evaluated the expression status of lncRNA POU3F3/miR-650/MGMT in melanoma tissues. Our results showed an increased MGMT expression in those with high lncRNA POU3F3 levels. A negative correlation between miR-650 and MGMT was also observed in melanoma tissues. Further studies also supported high lncRNA POU3F3 levels in the disease progression of patients with melanoma who received the DTIC-based chemotherapy. Cox-regression and survival analysis indicated that the expression of lncRNA POU3F3 was a detrimental factor for the progression of melanoma. The patients with lncRNA POU3F3-positive melanoma showed a shorter survival time than other patients. Our results supported a promising survival estimation value of lncRNA POU3F3 in patients with melanoma.

Our study suggested that the lncRNA POU3F3/miR-650/MGMT pathway is a novel target for improving the therapeutic efficiency of alkylating agents-based chemotherapy for melanoma. Given that the limited survival time was calculated for patients in the late stage of melanoma and the increasing trends of the incidence, further clinical trials for an lncRNA POU3F3-targeted therapy is considered as a promising strategy for melanoma treatment.

## Data Availability Statement

The original contributions presented in the study are included in the article/Supplementary Material, further inquiries can be directed to the corresponding author/s.

## Ethics Statement

The animal study was reviewed and approved by the Animal Care and Use Committees of PLA 960th Hospital.

## Author Contributions

KW, QW, and S-LL were involved in the concept and design of the study. KW, QW, Y-LL, ZX, and Q-QW contributed to acquiring the data. KW, QW, LY, and S-LL contributed to the analysis and interpretation of the data. KW and S-LL drafted the manuscript. All authors revised the manuscript and approved the final version.

## Conflict of Interest

The authors declare that the research was conducted in the absence of any commercial or financial relationships that could be construed as a potential conflict of interest.
