# Supplementary material for: LncRNA POU3F3 Contributes to Dacarbazine Resistance of Human Melanoma Through the MiR-650/MGMT Axis
Source: Front Oncol. 2021 Mar 17;11:643613. doi: 10.3389/fonc.2021.643613 (PMC8010678; doi:10.3389/fonc.2021.643613)
Supplement: Supplementary file 1 [file Data_Sheet_1.docx]

**Supplemental data**


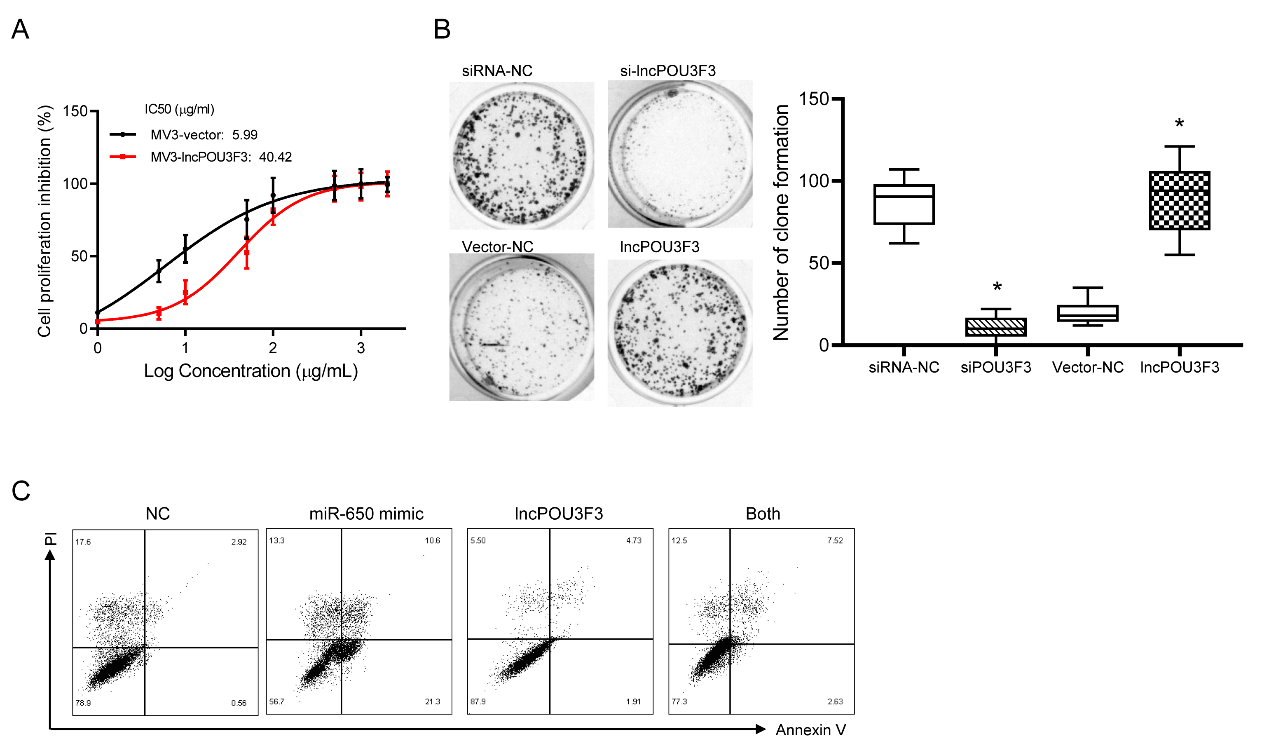


Supplemental Figure 1. LncRNA POU3F3 promotes MV3 cell proliferation with DTIC treatment.

(A) Transfected MV3 cells were treated with a series dose of DTIC (1-2000 μg/ml) for 48h, and cell viability was determined with MTT assays. IC50 was analyzed with the percentage of cell viability inhibition. (B) Totally 1000 transfected DTIC-resistant and parental MV3 cells were cultured with DTIC for 10 days. Cell colonies were compared between different transfected cells. (C) Cells were co-infected with lncRNA POU3F3 and miR-650 or not and then treated with DTIC for 48h. The cell apoptosis percentage was analyzed with flowcytometry. The data are presented as mean ± SD of at least three independent experiments. * P < 0.01.

**Supplemental Table 1**. Primers sequences in qRT-PCR assays.

| RNA | Primer sequences |
| --- | --- |
| LncRNA POU3F3 | Forward, 5’-TCCGCTAAGTGGTCTCACAG-3’  Reverse, 5’-CCCAGGAATCCATAGACTGC-3’ |
| miR-650 | Forward, 5’-AGAGGAGGCAGCGCTCT -3’  Reverse, 5’-CAGTGCGTGTCGTGGAGT-3’ |
| MGMT | Forward, 5’-TTTTCCAGCAAGAGTCGTTCAC-3’  Reverse, 5’-GGGACAGGATTGCCTCTCAT-3’ |
| GAPDH | Forward, 5’-ATCCCATCACCATCTTCC-3’  Reverse, 5’-GAGTCCTTCCACGATACCA -3’ |
| U6 | Forward, 5’-CTCGCTTCGGCAGCACA-3’  Reverse, 5’-AACGCTTCACGAATTTGCGT-3’ |
